# A two-step process for *in silico* screening to assess the performance of qRTPCR kits against variant strains of SARS-CoV-2

**DOI:** 10.1186/s12864-022-08999-3

**Published:** 2022-11-17

**Authors:** Swati Gupta, Amit Kumar, Nivedita Gupta, Deepak R. Bharti, Neeraj Aggarwal, Vasanthapuram Ravi

**Affiliations:** 1 Division of Epidemiology and Communicable Disease, V. Ramalingaswami Bhawan, Indian Council of Medical Research, Ansari Nagar, New Delhi, 110029 India; 2 Biomedical Informatics Division, Indian Council of Medical Research, Ansari Nagar, New Delhi, India; 3 Department of Forensic Medicine & Toxicology, All India Institute of Medical Sciences, Ansari Nagar, New Delhi, India; 4 Department of Neurosciences, National Institute of Mental Health and Neuro-Sciences, Bengaluru, 560029 India

**Keywords:** Delta, Omicron, SARS-CoV-2 variant, *In silico* analysis, qRT-PCR kits

## Abstract

**Background:**

Since inception of the COVID-19 pandemic, early detection and isolation of positive cases is one of the key strategies to restrict disease transmission. Real time reverse transcription polymerase chain reaction (qRTPCR) has been the mainstay of diagnosis. Most of the qRTPCR kits were designed against the target genes of original strain of SARS-CoV-2. However, with the emergence of variant strains of SARS-CoV-2, sensitivity of the qRTPCR assays has reportedly reduced. In view of this, it is critical to continuously monitor the performance of the qRTPCR kits in the backdrop of variant strains of SARS-CoV-2. Real world monitoring of assay performance is challenging. Therefore, we developed a two-step *in-silico* screening process for evaluating the performance of various qRTPCR kits used in India.

**Results:**

We analysed 73 qRT-PCR kits marketed in India, against the two SARS-CoV-2 VoCs. Sequences of both Delta (B.1.617.2) and Omicron (B.1.1.529) VoCs submitted to GISAID within a specific timeframe were downloaded, clustered to identify unique sequences and aligned with primer and probe sequences. Results were analysed following a two-step screening process. Out of 73 kits analysed, seven were unsatisfactory for detection of both Delta and Omicron VoCs, 10 were unsatisfactory for Delta VoC whereas 2 were unsatisfactory for only Omicron VoC.

**Conclusion:**

Overall, we have developed a useful screening process for evaluating the performance of qRTPCR assays against Delta and Omicron VoCs of SARS-CoV-2 which can be used for detecting SARS-CoV-2 VoCs that may emerge in future and can also be redeployed for other evolving pathogens of public health importance.

**Supplementary Information:**

The online version contains supplementary material available at 10.1186/s12864-022-08999-3.

## Introduction

Severe acute respiratory syndrome coronavirus 2 (SARS-CoV-2) is a betacoronavirus with RNA strand size of ~ 29.9 kb [1]. After release of its genome sequence by China CDC on January 12, 2020 [2], several nucleic acid amplification tests (NAAT) have been developed for virus detection. Among the available NAATs, real-time reverse transcription polymerase chain reaction (qRT-PCR) is considered to be the gold standard test for detection of SARS-CoV-2 RNA in clinical samples [3]. Common genes of SARS-CoV-2 targeted for the development of qRT-PCR kits include Envelop (E), Nucleocapsid (N), Spike (S), Membrane (M) and Open Reading Frames (ORFs) i.e. ORF1 ab which also contains the gene encoding for RNA dependent RNA polymerase (RdRP/nsp12) [4]. As the COVID-19 pandemic evolved, the SARS-CoV-2 virus accumulated multiple mutations, most abundant being in the S gene, thus giving rise to variants of concern (VoCs) and variants of interest (VoIs) [5].

S gene encoded spike protein is located on the surface of the virus, abundantly expressed and facilitates virus entry into the host cell. Earlier in the pandemic, S gene target was increasingly selected for inclusion in the molecular diagnostic tests, due to high sensitivity and specificity of detection of this gene. Later, due to the high mutation rates observed in the S gene, other genes having conserved sequences and relatively lesser rates of mutation were preferred [6]. Diagnostic kits with two or more target genes are now increasingly being used to improve the sensitivity of qRT-PCR assays.

At times, mismatch of even a single nucleotide can drastically affect the performance of a test kit. Mutations in regions specific for the hybridization of primers and probes affects the primer-probe binding efficiency, thus giving false negative or inconclusive results. SARS-CoV-2 Alpha /B.1.1.7 variant, Delta /B.1.617.2 variant and Omicron /B.1.1.529 variant had several mutations in the S gene, due to which S gene target failure (SGTF) or S gene drop out in qRT-PCR tests was observed with Alpha and Omicron VoCs [7–9]. However, despite the mutations in S gene, B.1.617.2 (Delta variant) had no SGTF [10]. Similarly, multiple mutations in N gene affected its detection and thus generated false negative RT-PCR results [11, 12]. Most of the available qRT-PCR tests were designed to have acceptable sensitivity and specificity against the early reported sequences of SARS-CoV-2. However, with the introduction of VoCs and VoIs, the sensitivity of these assays has reportedly reduced [13].

It is therefore imperative to monitor the performance of available NAAT tests in the backdrop of emerging VoCs and VoIs. Here we describe the development of a two-step screening process to assess the performance of existing qRT-PCR test kits for their ability to detect the circulating Delta and Omicron variants across the world, using an *in silico* approach. The screening process developed by us can be used for assessing the utility of various NAAT tests against the SARS-CoV-2 variants in future.

## Methodology

### Primer and probe sequence retrieval

Since inception of the COVID-19 pandemic, the Indian Council of Medical Research (ICMR) was designated as the nodal agency for validation of the qRT-PCR diagnostic test kits for SARS-CoV-2 in India. Earlier in the pandemic, manufacturers were not mandated to submit their primer probe sequences to ICMR, when applying for validation. Later on, due to the repeated emergence of SARS-CoV-2 VoCs and VoIs, there was a critical need to assess the performance of various test kits already marketed in India. Therefore, ICMR contacted the manufacturers of already approved and marketed qRT-PCR kits and requested them to share the primer and probe sequences. Subsequently it was made mandatory for all qRT-PCR kit manufacturers to submit the primer-probe sequences at the time of making a validation request to ICMR.

### Kit selection and database creation

Out of the 200 companies contacted retrospectively, 76 kit manufacturers reverted back with the sequence of their primers and probes. Out of these, only 73 kits were selected for analysis as kits based on LAMP assay and having spacer in their primer and probe sequences were excluded. A database of all the selected kits was prepared with details of name of the kit, name of the manufacturer, target genes and length of the primer/probes along with their sequences. All selected kits were anonymized and primer/probe sequences were converted into FASTA format for further analysis.

### SARS-CoV-2 whole genome sequence (WGS) dataset retrieval

Complete genome sequences with less than 1% ambiguous bases (Ns) of Delta and Omicron VoC of SARS-CoV-2 were downloaded from global initiative on sharing all influenza data (GISAID) database [14–16]. For downloading Delta sequences available in GISAID, keywords Delta, time-period till July 15, 2021, complete sequences (less than 1% ambiguous bases (Ns)) and high coverage were used. For downloading Omicron sequences, keywords, Omicron, time-period till January 19, 2022, complete sequences (less than 1% ambiguous bases (Ns)) and high coverage were used. Incomplete sequences were excluded. A total number of 186,355 and 392,855 complete sequences of Delta and Omicron were included in this study respectively.

### Removal of repeat sequences and identification of unique sequences

To spot identical sequences of Delta and Omicron VoCs, all selected genome sequences were clustered using CD-HIT software [17] with sequence identity cut-off equal to 1.0 (other parameters were left at default settings). Unique sequences thus obtained were selected for further analysis/ BLAST search [18]. Similarly, all primer and probe sequences were also subjected to CD-HIT clustering for selection of unique sequences and further BLAST search. Since BLAST search is a computation expensive program, this method was used to reduce computational time.

### SARS-CoV-2 genome alignment with primers and probes

Selected unique genome sequences were used to create a database for local BLAST search using “makeblastdb” command. Primer and probe sequences were searched in the database using “blastn” program of BLAST. All the above analysis were done on Intel Xeon Gold server with 64 processor and 256 GB RAM.

**Fig. 1:**
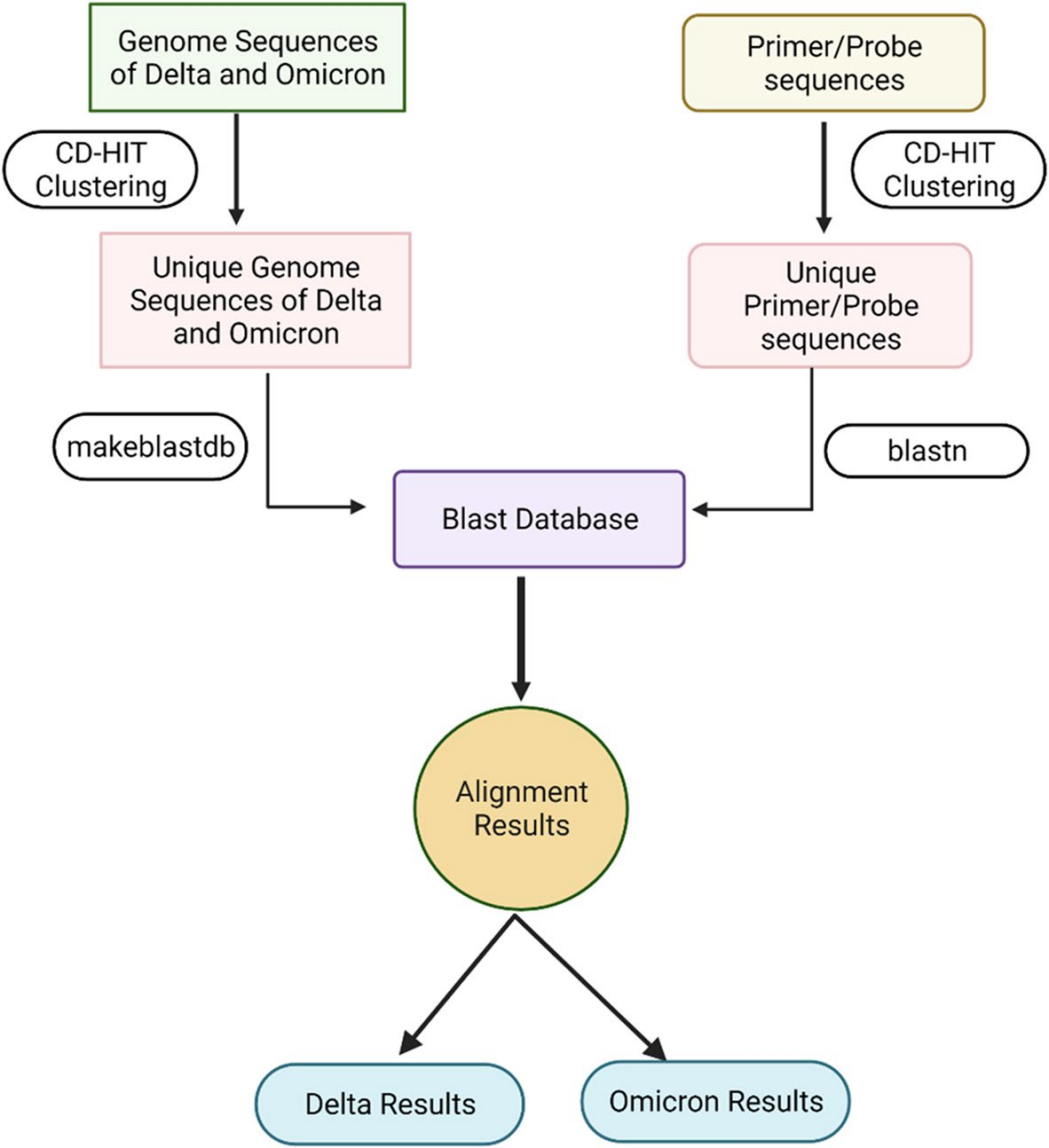
Flow chart for primer and probe data processing. Details provided in SARS-CoV-2 genome alignment with primers and probes

Figure 1 represents the process of database creation. Results were parsed and a comparative table was generated for further analysis. Pairwise alignment of each forward & reverse primer and probe sequence with the full genome sequences of the two VoCs were retrieved under the following categories:

- Percentage of alignment of each primer/probe with the selected VoCs of SARS-CoV-2.
- Length of the primer/probe sequence match with the full genome of SARS-CoV-2, against the total length of the primer/probe.
- Number of mismatches and gaps between the primer/probe and the VoC sequence on their alignment.
- 5′ and 3′ start and end location of primer/probe alignment with the VoC sequences.

### Mismatch identification

To obtain the positions of mismatches in primers and probes, EMBOSS Water (EMBOSS: 6.6.0.0) package [19] was used. EMBOSS Water is a modified version of the Smith-Waterman algorithm [20] for local alignment. Results were parsed to obtain the mismatched positions for each database entry and later summarised to obtain position-wise mismatches across the length of the primers and probe.

### Two-step screening process for assessing the performance of qRT-PCR kits by *in silico* approach

Based on the available evidence and published literature [21–27], a two-step screening process was developed. This two-step screening processis the first of its kind where all the parameters for screening of primers and probes have been included. In the first level screening, complete global sequences of VoC submitted to GISAID were considered and subsequently, in the second level screening, complete sequences submitted to GISAID from India were analysed.

Following were the screening criteria used in analysis:

#### First level screening criteria

i. Each primer or probe sequence (irrespective of the length) under review, must exhibit at least 95% alignment with the genome sequences of the VoC, downloaded from GISAID.
ii. Full length of the primer or probe must match with at least 95% of the sequences with which they aligned in step (i) of screening.
iii. Even a single mismatch at 3′ or 5′ ends was considered unacceptable [21, 23–26].
iv. Missing or mismatch of three contiguous nucleotides in primer/ probe alignment was considered unacceptable [27].
v. A single nucleotide mismatch in the middle of the probe was considered unacceptable. Therefore, for both odd and even number nucleotides in the probe, central location i.e. -1, 0, + 1 nucleotides must align with the genome sequence [21, 25].
vi. Notwithstanding any of the above, primers/probes having degenerate nucleotides were considered acceptable if even a single combination of nucleotide meets all the above criteria.

#### In addition to the above criteria, the following decision-making matrix was developed for assessing the *in silico* performance of testing kits

- E gene was considered as screening whereas S, N, ORF, M and RdRp were considered as confirmatory targets. In addition, for single S gene assays, S gene was considered as confirmatory and in two or more gene assay it was considered as a screening gene. For the two gene assays, wherein E and S genes were targets, the S gene was considered as confirmatory [28, 29].
- A strategy was developed to identify the centre of the probe. E.g. for probe with odd number of nucleotides, e.g. 11 nucleotides, nucleotide at number six was considered at central location (0) and − 1 and + 1 nucleotides were at 5 and 7 location respectively. For probes with even number of nucleotides, e.g. 12 nucleotides, 6.5 was considered as the central location and therefore nucleotides at position 6 and 7 which are − 1 and + 1 were considered for detecting mismatch location.
- Kits with two or more gene targets were assessed on the following basis:
  Primers and probes of each individual gene target to essentially meet the criteria i to v and vi, if applicable.
  For kits using a combination of both screening and confirmatory genes, if the confirmatory gene met the criteria i to v and vi, if applicable, irrespective of the performance of the screening gene target, the kit performance was labelled as satisfactory.
- For kits using a combination of both screening and confirmatory genes, if the confirmatory gene did not meet the screening criteria i to v and vi, if applicable, the kit was labelled as unsatisfactory irrespective of the performance of the screening gene target.

#### Second level screening criteria

Primers or probes found to be unsatisfactory as per criteria number (i) and (ii) in the first level screening analysis, were subjected to second level screening analysis to rule out the possibility due to huge number of global sequences submitted to GISAID. Sequences having mismatch at 3′ or 5′ ends or probes having mismatch at central location i.e. -1, 0, + 1 nucleotides were rejected and were not considered for reanalysis. For reconfirmation, representative complete genome sequences of SARS-CoV-2 submitted to GISAID from India were selected for reanalysis. Criteria (i) to (vi) of second level screening analysis were the same as those used for the first level screening analysis except for the difference of use of only Indian VoC.

The second level screening provided advantage to the kits that did not qualify in first level screening due to stringent criteria of 95% alignment with total number of downloaded sequences and 95% length wise alignment with downloaded sequences. Sequences submitted from India were also part of first level screening but huge number of global sequences resulted in rejection of some kits based on 95% criteria.

### Selection of representative SARS-CoV-2 genomes from India

Complete genome sequences with less than 1% ambiguous bases (Ns) submitted to GISAID from India were downloaded for Delta with keywords Delta, time-period till July 15, 2021, complete sequences (less than 1% ambiguous bases (Ns)), high coverage and India. For downloading Omicron sequences, keywords, Omicron, time-period till January 19, 2022, complete sequences (less than 1% ambiguous bases (Ns)), high coverage and India, were used. A total of 17,238 Delta and 7994 Omicron genome sequences were downloaded. These sequences were selected for second level screening. Subsequently, clustering was done to group the identical sequences and identifying the unique sequences using the CD-HIT software. The primers or probes which could not meet the second level screening analysis were finally considered as unsatisfactory.

## Results

### qRT-PCR kit types based on the number of SARS-CoV-2 target genes

Data obtained from qRT-PCR kit manufacturers was classified into four groups based on the number of genes used for detection of SARS-CoV-2. Kits having one set of primers and probes for SARS-CoV-2 gene detection were considered as single gene kits. Similarly, two, three and four gene kits had two, three and four sets of primers and probes respectively for SARS-CoV-2 target gene detection. A total of 3, 53, 16 and 1 kits were in the one, two, three and four gene kit category respectively.

### Number of unique sequences of SARS-CoV-2 identified for first and second level screening analysis

**Fig. 2:**
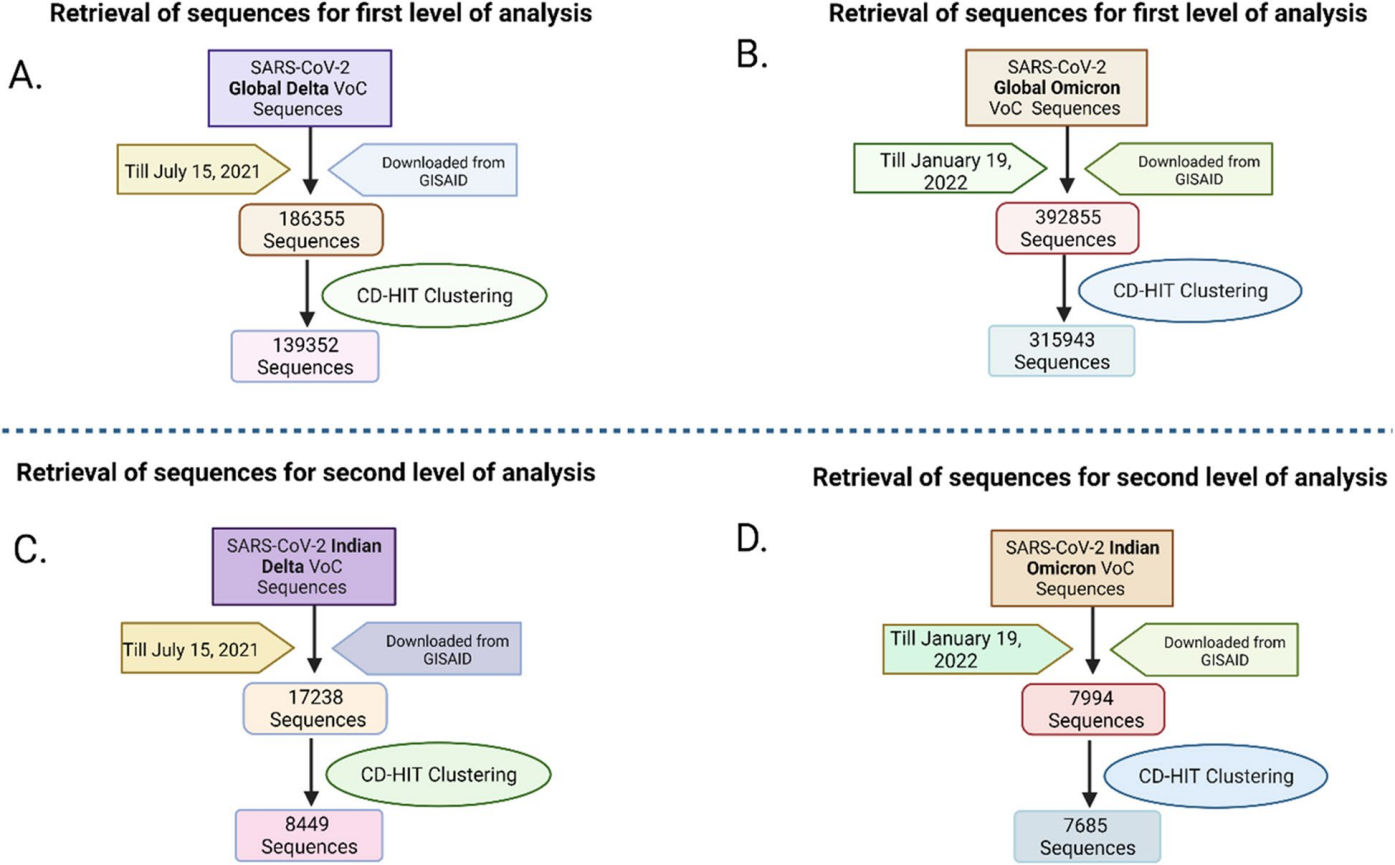
Flow chart describing the process of identification of unique sequences of Delta (**A**&**C**) and Omicron (**B**&**D**) among global and Indian SARS-CoV-2 sequences, downloaded from GISAID, for first (**A **&** B**) and second (**C **&** D**) level screening analysis

For the first level screening analysis, a total of 1,86,355 Delta and 3,92,855 Omicron genome sequences were downloaded from GISAID. On clustering, 1,39,352 and 3,15,943 unique sequences were identified for Delta and Omicron respectively. There sequences were finally used to create database for local BLAST search (Fig. 2A and B).

For the second level screening analysis, a total of 17,238 Delta and 7994 Omicron genome sequences were downloaded. On clustering, 8449 and 7685 unique sequences were identified for Delta and Omicron, respectively. Figure 2 describes the process of clustering followed for identification of unique sequences of Delta and Omicron VoCs for the first (Fig. 2A and B) and second level screening analysis (Fig. 2C and D).

#### Results of the first and second level screening analysis

Primer & probe alignment data for both Delta and Omicron VoCs was obtained and analysed by applying the first level screening criteria. Results of the analysis are summarized in Table 1. The standalone four gene kit was found to perform satisfactorily for detection of both VoCs. 81% of three gene and 74% of two target gene kits were found to be satisfactory for detection of the Delta VoC, whereas 81% of three and 64% of two target gene kits were found to be satisfactory for detection of the Omicron VoC.

**Table 1:** A describes the *in silico* qRT-PCR kit validation with SARS-CoV-2 Delta variant and B describes the in silico qRT-PCR kit validation with SARS-CoV-2 Omicron variant

A							
***In silico*** **evaluation of qRT-PCR Kits with SARS-CoV-2 Delta Variant**							
	**Alignment results with global Delta sequences (First level screening analysis)**			**Alignment results with Indian Delta sequences (Second level screening analysis)**			**Overall results**^**a**^
**Type of Kit**	**Total number of kits**	**No. satisfactory**	**% satisfactory**	**No. of kits**	**No. satisfactory**	**% satisfactory**	**Overall satisfactory %**
One gene	3	2	67	0	NA	NA	67
Two gene	53	39	74	1	1	100	75
Three gene	16	13	81	2	0	0	81
Four gene	1	1	100	0	NA	NA	100
Total	73	55	75	3	1	33	77
B							
***In silico*** **evaluation of qRT-PCR Kits with SARS-CoV-2 Omicron Variant**							
	**Alignment results with global Omicron sequences (First level screening analysis)**			**Alignment results with Indian Omicron sequences (Second level screening analysis)**			**Overall results**^**a**^
**Type of Kit**	**Total number of kits**	**No. satisfactory**	**% satisfactory**	**No. of kits**	**No. satisfactory**	**% satisfactory**	**Overall satisfactory %**
One gene	3	1	33	2	1	50	67
Two gene	53	34	64	16	12	75	87
Three gene	16	13	81	2	2	100	94
Four gene	1	1	100	0	NA	NA	100
Total	73	49	67	20	15	75	88

^a^The overall results were calculated by adding the number of satisfactory kits in first and second level screening then divided by total number of kits in each category and expressed as percentage

Further, after application of the second level screening criteria, the overall satisfactory alignment percentage for two target gene kits increased to 75 and 87% for Delta and Omicron sequences, respectively. Whereas, for three target gene kits for Omicron VoC the satisfactory alignment increased to 94%. However, there was no change for Delta VoCs for three target gene kits.

Overall, irrespective of the number of target genes, by applying the two-step screening process, 77 and 88% of already marketed RTCPR kits met the *in silico* screening criteria for achieving a satisfactory score for Delta and Omicron VoCs, respectively.

#### Criteria for classifying qRT-PCR kits as unsatisfactory

**Fig. 3:**
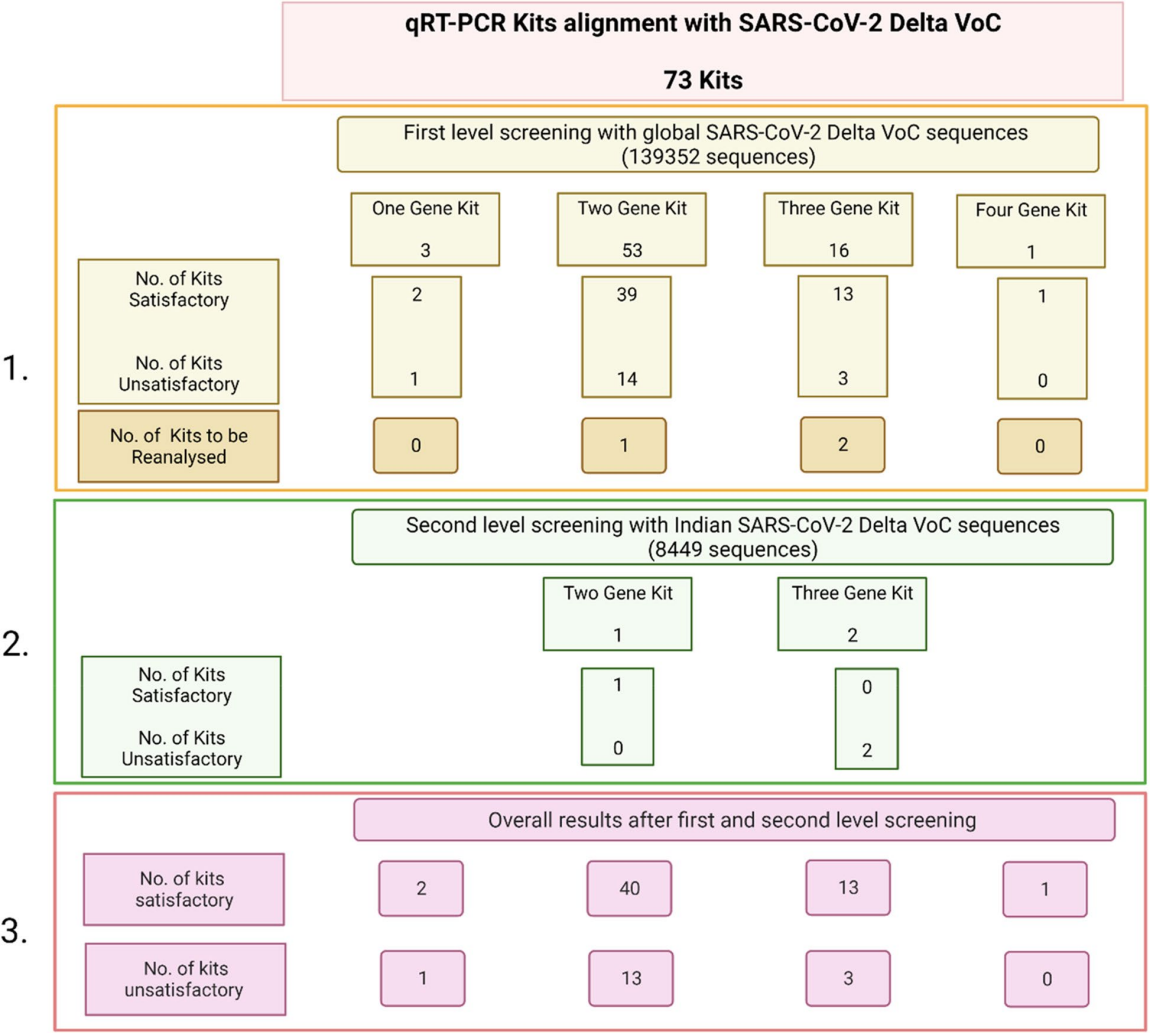
Number of kits found satisfactory and unsatisfactory in first (1.) and second (2.) level screening and overall number (3.) of satisfactory and unsatisfactory kits on alignment with global and Indian Delta VoC sequences of SARS-CoV-2 after applying first and second level screening

With Delta VoC sequences, 18 out of 73 (25%) kits were found to be unsatisfactory after alignment with global Delta sequences, with majority of them found unsatisfactory on the basis of a single common criteria of missing alignment of primers and probes at 3′ or 5′ end i.e. criteria iii of first level screening analysis (Fig. 3). Three kits were subjected to second level screening analysis with Indian Delta sequences as they were found unsatisfactory as per criteria (i) and (ii) of the first level screening analysis. Two out of three (67%) kits were found to be unsatisfactory after second level screening. In majority of cases, RdRp, N and ORF gene primers and S, ORF and RdRp gene probes did not align with Delta VoC as per the two step screening criteria (Supplementary data file).

**Fig. 4:**
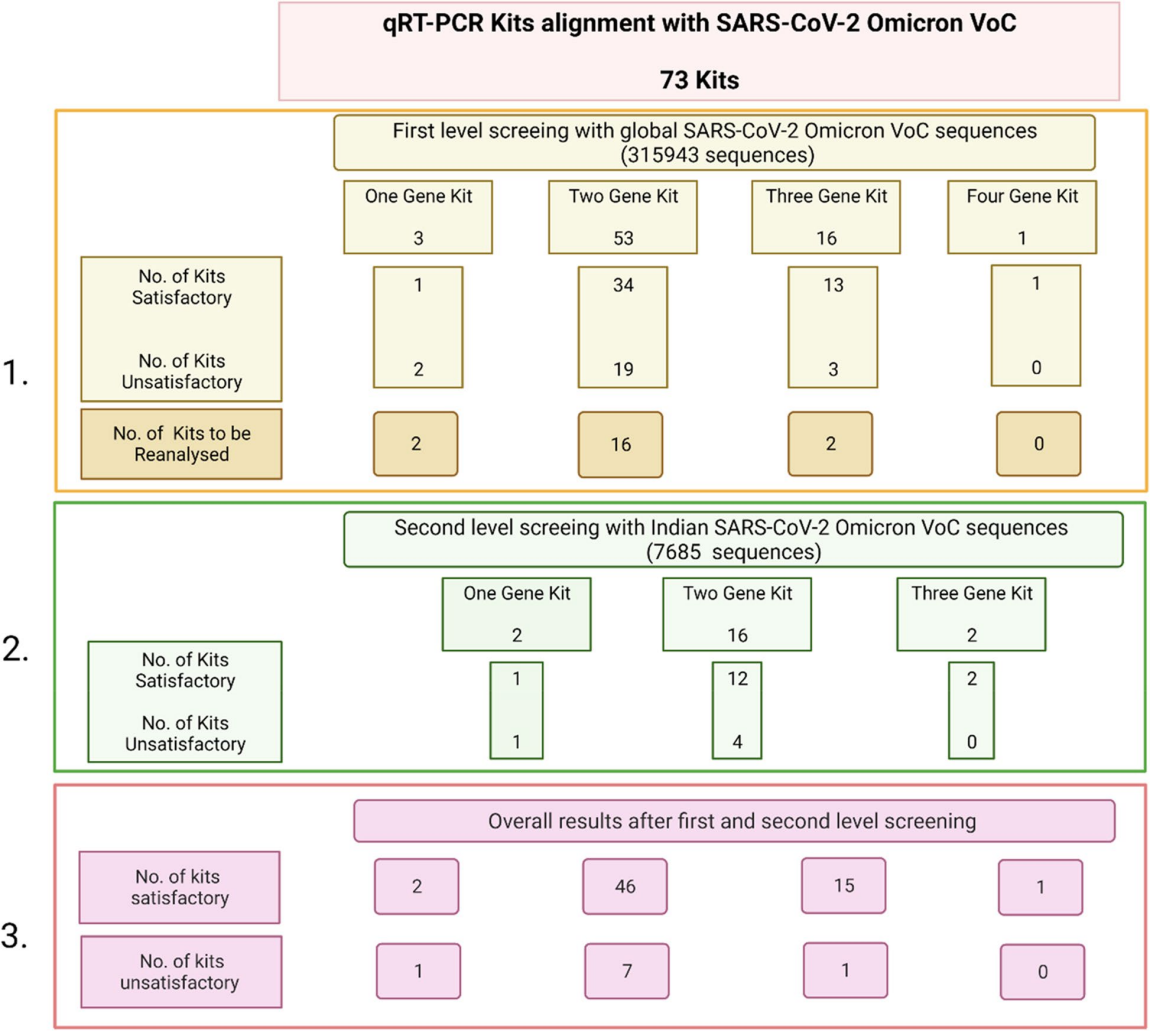
Number of kits found satisfactory and unsatisfactory in first (1.) and second (2.) level screening and overall number (3.) of satisfactory and unsatisfactory kits on alignment with global and Indian Omicron VoC sequences of SARS-CoV-2 after applying first and second level screening

For Omicron VoC, 24 out of 73 (33%) kits were found to be unsatisfactory in the first level screening, of which 4 kits were rejected as there was no alignment of primers and probes at 3′ or 5′ end. Subsequently, 20 kits underwent second level screening analysis with Indian Omicron sequences as they were found unsatisfactory as per criteria (i) and (ii) of the first level screening analysis. A total 5 out of 20 (25%) kits were found to be unsatisfactory after second level screening. Out of five, three kits were rejected as they failed to meet criteria i and ii and two kits could not meet criteria iii of second level screening analysis (Fig. 4). In majority of cases, S, N and E gene primer and ORF, S, N and RdRp gene probe did not align with Omicron VoC as per the two step screening criteria (Supplementary data file).

Overall, 17 out of 73 (23%) kits were found to be unsatisfactory on alignment with Delta sequences of SARS-CoV-2 and 9 out of 73 (12%) kits were found unsatisfactory on alignment with Omicron sequences of SARS-CoV-2. Out of 9 kits found unsatisfied to detect Omicron, seven kits were found unsatisfactory to detect Delta variant also.

## Discussion

With time, SARS-CoV-2 has evolved into variants of concern which have enabled the virus to escape the host immune response, enhance its transmissibility and evade diagnosis by standard methods. One of the primary reasons for diagnostic evasion is the occurrence of mutations in the target region leading to non-hybridization of primers/probes. The pathognomic feature of the B.1.1.7 or alpha VoC, identified first in UK, was S gene target drop-out in multiplex assays [9, 30]. S gene failure was not observed much with the Delta VoC [31], however with emergence of Omicron VoC, S gene target failure was again consistently seen with BA.1.1.529 [32]. In addition, N gene failure was also reported with the Omicron VoC [33]. However, again with the emergence of sublineages of Omicron VoC, the S and N gene target failures became inconsistent and were largely dropped as screening methods for these VoCs [33].

Since inception of the pandemic, early detection, contact tracing and containment of infection were globally identified as key strategies for preventing transmission and controlling spread of the disease. However, over the past 1 year, the focus has shifted towards isolation of infected patients for preventing disease spread to vulnerable individuals and eventually reducing hospitalizations and deaths. In both situations, the role of reliable diagnostics is pivotal. Early detection of infected individuals and their isolation can only be achieved if the diagnostic tests are accurate and have high sensitivity and specificity for detecting the variant strains. Since majority of tests for detection of SARS-CoV-2 are based on target genes of conventional strains of the virus, their performance has been questioned in the backdrop of emergence of SARS-CoV-2 VoCs.

Early in the pandemic, single-plex kits were recommended by WHO for use in regions with established transmission of SARS-CoV-2 [34]. Singleplex kits are known to have a better sensitivity as compared to multiplex assays [35], with the trade-off of missing diagnosis on emergence of variant strains of SARS-CoV-2. While single S gene target based kits are likely to miss diagnosis in a significant number of clinical samples, dual-, triple- or multiplex kits with two or more primers/probes directed against the target genes of the virus, reduce chances of RTPCR failure and improve accuracy [36, 37]. Therefore, the use of singleplex assay was not preferred later in the pandemic, as the public health strategy has always necessitated the use of sensitive assays.

With the evolution of SARS-CoV-2 virus it is critical to continuously monitor the performance of existing molecular assays with time. Assessing the performance of each diagnostic assay in field is labour-intensive and challenging. Thus, it is desirable to develop simple *in silico* process to reliably estimate the performance of different molecular assays in field. *In silico* analysis of the binding efficiency of primers and probes with the VoC sequences of SARS-CoV-2, submitted to available public databases like GISAID is a feasible method to reliably estimate the performance of various assays.

In the present study we have developed a two-step *in silico* screening process for estimating the performance of several qRTPCR kits used in India. *In silico* evaluation of qRT-PCR kits has been undertaken in other studies also [22, 23]. However, none of the studies had proposed a standard process considering all the parameters for assessing the impact of circulating or new VoC on performance of molecular assays. In addition, a strategy to address the challenge of locating the centre of probes with odd and even number of nucleotides was also developed which is one of its own kind. Further, the primer or probes having degenerate nucleotides satisfying all the screening criteria with even a single nucleotide combination were given preference. Moreover, the data on mismatch location of a specific primer or probe provide the guidance to the kit manufacturers for redesign at particular location. Also the data of concerning/not aligning primers and probes guide the country to not consider the kits for diagnosis of VoC having sequence similar to the concerning ones.

Overall, our study has several strengths. Besides evaluating the performance of each primer and probe, our two-step screening process has also taken into consideration the sub-optimal performance of kits not satisfying the stringent 95% screening criteria i.e. 95% alignment with the number of downloaded sequences and 95% lengthwise alignment with downloaded sequences, mentioned as criteria (i) and (ii). Alignment of each primer and probe used in India, to the large number of global sequences may also be misleading at times due to difference in SARS-CoV-2 strains circulating globally. Therefore, alignment with lesser number of country specific sequences (with less number of 95% downloaded sequences) gave opportunity to the kit manufacturers by re-evaluating their kits based on the circulating VoC in the given country.

Also, this two-step screening process can help the countries in balancing the risk of epidemiologically missing the cases for a particular variant not pre-dominantly circulating over the pre-dominantly circulating variant of SARS-CoV-2. This improved the stringency of screening.

Our study has a few limitations also. Though the screening process developed by us in the present study has considered various possibilities for evaluation of qRTPCR kits for detection of SARS-CoV-2, but *in silico* approach needs to be further validated by real word performance. Similarly, multiple softwares are available for clustering and alignment of primers/probes with the downloaded sequences. We could not compare these softwares and provide specific recommendations on the most suitable ones to be used. Also, we could only retrieve primer/probe sequences from 73 kit manufacturers out of 200 kit manufacturers contacted. Thus we were not able to evaluate the overall performance of all qRT-PCR kits available in Indian market.

## Conclusion

Overall, we have developed a useful process for screening the performance of qRTPCR assays for detection of SARS-CoV-2. This process has been standardized against Delta and Omicron VoCs, however it has the potential of use for other variants of SARS-CoV-2 that may emerge in future. The two-step screening process developed by us also has the potential to be used for other evolving pathogens of public health importance.

## Supplementary Information

**Additional file 1.**

## Data Availability

The unidentified raw data supporting the conclusions of this article will be made available by the corresponding author without undue reservation.

## Abbreviations

SARS-CoV-2: Severe acute respiratory syndrome coronavirus 2
NAAT: Nucleic acid amplification test
E: Envelop
ICMR: Indian Council of Medical Research
N: Nucleocapsid
S: Spike
M: Membrane
ORFs: Open Reading Frames
RdRP: RNA dependent RNA polymerase
VOCs: Variant of Concerns
VOIs: Variants of Interest
qRT-PCR: Real-Time Reverse Transcription Polymerase Chain Reaction
SGTF: S gene target failure
WGS: Whole Genome Sequence
